# Evaluation of adherence to anti-osteoporosis treatment from the socio-economic context

**Published:** 2015

**Authors:** M Abobului, F Berghea, V Vlad, A Balanescu, D Opris, V Bojinca, A Kosevoi Tichie, D Predeteanu, R Ionescu

**Affiliations:** * “Sf. Maria” Clinical Hospital, Bucharest, Romania

**Keywords:** anti-osteoporosis treatment, socio-economic context, osteoporosis, elder patients

## Abstract

Osteoporosis is a disease characterized by the reduction of the bone mass and the modification of the bone architecture, which leads to the risk of fracture of the fragile bones, this being the main clinical consequence of the disease. At the same time, osteoporosis is not only a problem by itself, but it is very important from the point of view of the consequences it may produce. Among its consequences, fractures should be mentioned especially in elders, their presence finally leading to an important decrease in the quality of life or even to death.

Osteoporosis affects a high amount of persons, preponderantly elders, being considered a very important problem as the society we are talking about deals with the problem of aging.

Socio-economical factors and their impact in the development of different pathologies have been seriously analyzed, especially by the western school of medicine.

The aim of the current study is to evaluate the adherence to the treatment for osteoporosis of patients diagnosed with osteoporosis or in whom this diagnosis was taken into consideration by the physician, according to some characteristics identified as being the most relevant by a group of specialists.

210 patients were evaluated in this study during January 2011 and December 2013.

This study highlighted the way patients with a real or presumptive diagnosis of osteoporosis adhere to the treatment for this disease according to the conditions considered relevant by a team of rheumatologists. It is important to notice that, still from the beginning, once the duration of the disease grows, patients become more and more conscious of the seriousness of the disease and more and more of them adhere to the treatment.

## Introduction

Osteoporosis is a disease characterized by the reduction of the bone mass and the modification of the bone architecture, which leads to the risk of fracture of the fragile bones, this being the main clinical consequence of the disease. At the same time, osteoporosis is not only a problem by itself, but it is very important from the point of view of the consequences it may produce. Among its consequences, fractures should be mentioned especially in elders, their presence finally leading to an important decrease in the quality of life or even to death. 

Osteoporosis affects a high amount of persons, preponderantly elders, being considered a very important problem as the society we are talking about deals with the problem of aging. Unfortunately, at the level of the European Union, generalized aging represents a problem especially in the old European Union, respectively in the western states and that is why a great amount of economical analyses and social impact evaluation of osteoporosis have been undergone in this field. In 2010, there were 22 million women and approximately 5,5 million men with osteoporosis in the European Union. In the same period, 3,5 million new fragility fractures were registered. Out of these, there were 620.000 hip fractures, 520.000 vertebral fractures, 560.000 forearm fractures and 1.800.000 of other fractures [**1**]. According to the same author, the costs of the incident fractures and of the anterior ones could be calculated in 2010 to approximately 37 billion euro, with a forecasting that in 2025, these costs would be higher with 25%.

Due to the fact that osteoporosis does not present any alarming signs or symptoms until the moment the fracture appears, it is understandable why the social costs and the economical impact are that important for persons who are diagnosed with osteoporosis *per primam*, in the moment they present to the physician for an osteoporotic fracture. These persons need a prolonged and sustained treatment so that the risk of fracture could be reduced. 

Socio-economical factors and their impact in the development of different pathologies have been seriously analyzed, especially by the western school of medicine. As far as osteoporosis is concerned, it should be taken into account that this entity has recently earned its title as an affection that can be identified and treated, and, as a result the socio-economical evaluations in osteoporosis are somehow late as compared to the ones of other pathologies. In a study undergone in 2008 [**4**], only 11 great socio-economical evaluations regarding the risk factors in osteoporosis have been identified. These socio-economical evaluations have been especially done according to some parameters such as: marital status, income, education, residential status (rural/ urban) and the occupation status. 

As far as the income is concerned, studies have shown that, until present, a great amount is inversely associated with a risk of osteoporotic fracture [**1**]. If we take into account the level of the private health insurances as a witness for the high level of incomes, we could evaluate that the low risk of fracture in osteoporosis in patients with a high level of private health insurances could be explained not only by the high level of incomes but also by a high interest for a better health state. Moreover, it is known that osteoporosis is connected to the way each patient promotes his/ her bone health state, the disease overwhelmingly benefiting from a prophylactic treatment before the cure.

Regarding the educational level and the risk of developing osteoporosis, studies had conflicting results; there is the study of doctor Wilson (Wilson, 2006) which showed that there is no osteoporosis risk associated to a lower educational level. On the other hand, there are also other studies (Vestergaard, 2006), which on the contrary have identified a converse risk of osteoporosis connected with the educational level in persons aged 40-59 years [**1**,**2**]. 

As far as the occupation status is concerned, Farahmand [**2**,**3**] identified a lower risk of osteoporotic fracture in persons who were employed as compared to the persons who were unemployed. However, the same results could not be identified in other studies undergone in the same period. 

If we take into account the residential status, the same study of Farahmand identified a lower risk of fracture in patients who lived in big houses compared to patients who lived in little houses. Still, it should be taken into account that the size of the house, respectively its surface, has a proportional connection with the level of the incomes. This way, the size of the house could be considered only an indirect indicator of incomes and respectively is a surrogate marker in the identification of risk factors in osteoporosis. Conflicting data also come from the evaluation of the marital status. In 2006 Wilson identified a lower risk of fracture in married persons compared to widow persons; still no association could be evidenced between osteoporosis, risk of fracture and married persons vs. persons who were never married. Espino and Yung (Espino 2000, Yung 2006) have also presented conflicting results [**2**].

## Objectives

The aim of the current study is to evaluate the adherence to the treatment for osteoporosis of patients diagnosed with osteoporosis or in whom this diagnosis was taken into consideration by the physician, according to some characteristics identified as being the most relevant by a group of specialists. 

## Methods & Patients 

210 patients were evaluated in this study during January 2011 and December 2013. These patients were selected through the method of consecutive selection of all the cases presented with the diagnosis of osteoporosis or to which a diagnosis of osteoporosis was taken into consideration in the above mentioned period. The patients consecutively presented to “Sfanta Maria” Hospital in Bucharest, a third hospital for the evaluation and treatment of osteoporosis. The inclusion criteria in this study were the following: diagnosis or the possible diagnosis of osteoporosis, adherence to the study conditions, respectively acceptance of study conditions by each patient and the acceptance to be included in the study, respectively the completion of at least 75% of the data in the questionnaire. The questionnaire was developed by a group of 7 rheumatologists with an experience of more than 5 years in treating osteoporosis. The realization of the questionnaire was done by a focus-group type of exercise in two stages. 20 criteria which had to be followed in these patients were established in the first stage. In the second stage, the criteria were reduced to 7 items, which were considered the most important for the objective of the study. The questionnaire also contained demographic data, data regarding the treatment of osteoporosis and of the rheumatism diseases which can modify osteoporosis, DXA evaluation and the age at the moment the questionnaire was completed. Bone mineral density was evaluated by a DXA examination which was considered important if it was performed at the level of the lumbar spine (L2-L4) or at the level of the femoral head, by using a device for bone densitometry approved for use in the European Union. The accepted precision of the device was of 0,75 ± 0,16%. The results were compared with the international standard accepted by the device, meaning the database of the device. In case the patients did not have a bone densitometry which could identify the diagnosis of osteoporosis, they were considered “patients at risk for osteoporosis” based on the following data: presence of vertebral fractures which were radiologically identified, presence of treatments considered to have generated osteoporosis in the context of rheumatism diseases (cortisone treatment for over 6 months, Metotrexat treatment for over 6 months, biological therapy for over 1 year), body mass index below the normal level according to the age and sex, and, in case of women, menopause appearance – naturally or surgically – below the age of 35 years. 

## Statistical analysis

The category variables were summarized in percents and the continuous ones were evaluated as medium and standard deviation when the data had a normal distribution. In order to evaluate the differences between the groups, chi square test, t-test, or the information offered by Wilcoxon test, were used. The results were considered important and statistically significant due to the fact that the value of p index was lower than 0,05 and the specific indices were higher than the standard values in literature. Data were analyzed by using the statistic program SPSS 16.0, developed in Chicago, U.S.A. 

## Results

**Fig. 1:**
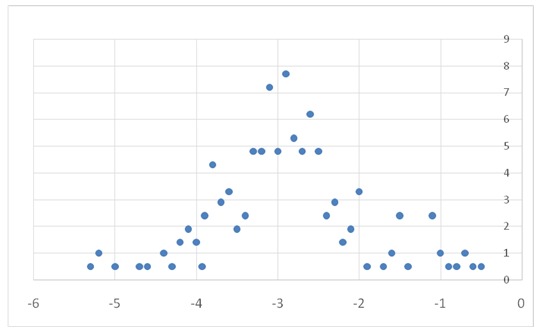
DXA value/ % of cases

210 patients who met the inclusion criteria of the study were identified during the period mentioned. Among these, 77% presented a DXA value lower than -2,5, which was defining for the diagnosis of osteoporosis. For 23%, the DXA evaluation did not identify the diagnosis of osteoporosis, despite the existence of the presumptive criteria of diagnosis. 73 patients already had a treatment for osteoporosis, as it can be seen in **Fig. 1**.

Age was medium, of 64,69 years, with a standard deviation of 11,4, having a maximum of 87 years and a minimum of 31 years. Among the patients diagnosed with osteoporosis, the duration from diagnosis to the inclusion in the study varied between minimum 1 year and maximum 16 years, respectively a standard deviation of 3,67 in a medium of 5,66 years. 108 patients were from Bucharest, respectively 51,4%, 66 patients were from the urban area, but from outside Bucharest, respectively 31,4% and 36 patients were from rural area, respectively 17,1%.

In 34 cases, respectively 16,2%, patients had 3 children or more; in 77 cases, respectively 36,7% they had 2 children; in 73 cases, respectively 34,8% they had 1 child and in 26 cases, respectively 12,4% they had no children. 

**Fig. 2:**
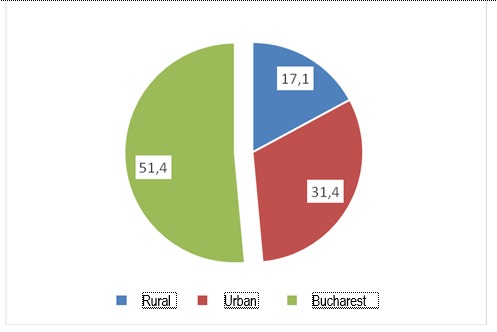
% of responders according to the residential status

**Followed treatments**

As mentioned before, 73 patients, respectively 34,8%, were under a treatment with biophosphonates for osteoporosis. 167 patients, respectively 79,5% were under a treatment in which a certain quantity of calcium was included, without it necessarily representing the optimum for the necessary according to age, sex and osteoporotic status. 48 patients, respectively 22,8% were under a treatment with corticoids, the dosage and the frequency were not important. 89 patients, respectively 42,4% of the cases were under a biological treatment. 169 patients, respectively 80,9% of the cases were under a treatment with non-steroid anti-inflammatory medicine or aspirin (which can be administered to patients who have heart problems). 81 patients, respectively 38,6% were under a treatment for coronary ischemic heart disease or hypertension.

Among the patients who were under corticoids treatment, only 43,6% were simultaneously under a treatment for osteoporosis with biophosphonates or similar for its prevention. As far as DXA evaluation was concerned, the value varied between -5,3 and -0,5 standard deviation, the data being available for 209 of the 210 patients. This way, the DXA evaluation had a medium value of -2,9 standard deviation, with a standard deviation of 0,88. 

**Fig. 3:**
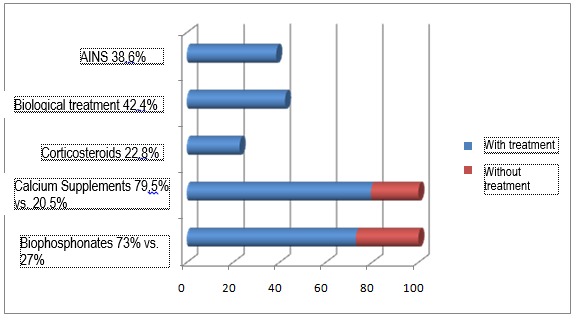
Treatments

**Evaluation of possible correlations**

Firstly, we were interested in the way the age of the patients was correlated with the age of the disease. This evaluation showed the way the patients selection criteria, met the conditions of quality of the study, as it is known that once they get older, the number of patients diagnosed with osteoporosis raises, just like the age of this diseases does. Pearson correlation between age and the age of the disease was of 0,608, highly correlated for p < 0,0001. This value certifies the quality of the analyzed lot of patients. Important correlations have also been met as far as age and adherence to treatment was concerned, respectively, older patients proved to adhere to treatment easier than younger patients. Although it did not have a high value (0,378), the Pearson correlation index in this case was highly specific (p <0,0001). A similar correlation, but of a higher intensity, was found between the age of osteoporosis and the adherence to osteoporosis, in the sense that an older age of the disease correlated with an index of 0,531, presenting a better adherence to treatment (p <0,0001). The elder patients had a positive answer to the question regarding the adherence to treatment and to the doctor’s advice (p <0,0001 for a correlation index of 0,340). Moreover, a superior correlation index (0,471) was identified between the way the patients listened to the doctor’s advice and the duration of the osteoporosis disease. An interesting correlation was observed regarding the way osteoporosis was perceived by the patients as a very severe disease or as an insignificant one. The correlation was significant (p < 0,007), negative (correlation factor of -0,187) between the perceived severity of the disease and age. This can be explained by the fact that as the patients get old, they perceive osteoporosis as a more serious disease. Moreover, the patients already diagnosed with osteoporosis had a more negative perception regarding the disease compared to the elder patients without a diagnosis of osteoporosis (correlation index of -0,233, p <0,001). As it was expected, the patients who considered that osteoporosis was a very serious disease were the ones who highly adhered to the treatment, respectively declared that they rarely did not follow the treatment for this disease (correlation index of -0,43, p <0,0001). As far as the way doctors perceive patients with osteoporosis are concerned, it can be observed that a very high correlation index 0,657 (p <0,0001), leads to the doctors’ possibility of identifying the patients who have adhered to the treatment. Furthermore, the doctors consider that patients adhere more to the treatment as the duration of the disease raises (correlation index of 0,364, p <0,0001). As far as age is concerned, there is a positive correlation, but of low amplitude, respectively p <0,0001 in a correlation index of 0,272, which can be explained by the fact that doctors do not consider age as a determinant factor for the adherence to the treatment. Doctors are able to identify these patients who consider osteoporosis a very serious disease. With a correlation index of 0,398 (p <0,0001), the doctors identify the patients who consider osteoporosis a serious disease or on the contrary an insignificant one. Moreover, the doctors easily manage to identify (correlation index of 0,563, p <0,0001) those patient who truly adhered to the treatment. Surprisingly, no correlation was identified between age and the way patients appreciate their quality of life (p <0,166), or between age and the satisfaction regarding the health state (p <0,649). Another interesting observation was that the patients who did not adhere to the treatment also had a negative feeling regarding the quality of life or the health state. This can be explained by a defeatist attitude of a particular group of patients (correlation index of 0,494, respectively 0,311, p <0,0001). At the same time, it is the connection between the way patients adhere to the treatment and the way they appreciate the quality of life, respectively the quality of health (correlation index of 0,386, respectively 0,232, p <0,001). However, patients who consider that they have a low quality of life or are unsatisfied with their health state do not consider this issue a result of the seriousness of osteoporosis. The correlation with the way osteoporosis is considered a serious or insignificant disease is reduced (correlation index of -0,224, respectively -0,062 in p <0,001). The doctors identify as being less compliant the patients who negatively appreciate their quality of life or are unsatisfied by the health state (correlation index of 0,717, respectively 0,398, p <0,0001).

**Fig. 4:**
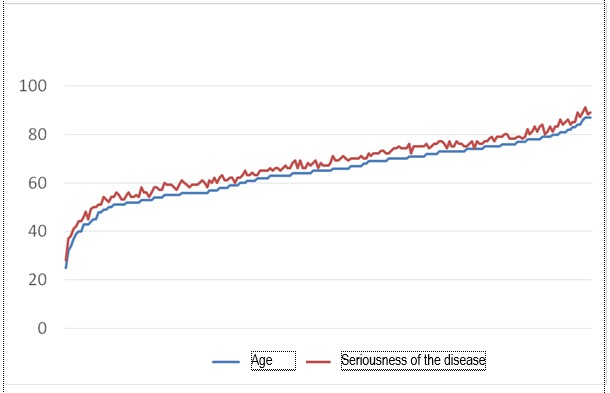
Correlation between the age of the patients and the perceived seriousness of the disease

Regarding the incomes, it was observed that there is no correlation between the incomes and the way they are appreciated, meaning subjectively, by the patient (the question was “What is your financial situation at present?”), the lack of correlation was registered at the level of the duration of diagnosis of osteoporosis or the adherence to the treatment (correlation index of 0,088, respectively 0,137, at p of 0,202, respectively 0,0047). This lack of correlation between the perceived financial capacity and the adherence to the treatment explains why patients do not consider the treatment for osteoporosis is mainly prevented by the access to high financial resources. Moreover, the doctors do not ascribe the adherence to the treatment to the financial situation of the patient either (correlation index of 0,105, p <0,131 – insignificant). It is interesting that the patients who consider they lack a good financial situation appreciate both their quality of life and the health state as being low.

**Fig. 5:**
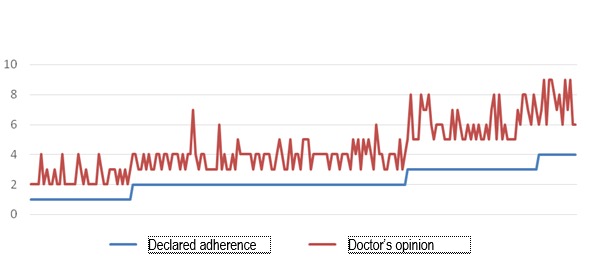
Correlations between the adherence to the treatment declared by the patient and the one evaluated by the doctor

## Discussions

The elder patients had a more positive answer to the question regarding the adherence to treatment and to the doctor’s recommendations. 

There is a correlation between the longer duration of diagnosis and the increase of adherence to treatment, explained by the fact that as the patients get old, they perceive osteoporosis as a more serious disease.

Doctors are able to accurately identify the patients who consider osteoporosis a very serious disease and those patients who are less compliant and negatively appreciate their quality of life or are unsatisfied by their health state.

This study highlighted the way patients with a real or presumptive diagnosis of osteoporosis adhere to the treatment for this disease according to the conditions considered relevant by a team of rheumatologists. It is important to notice that, still from the beginning, once the duration of the disease grows, patients become more and more conscious of the seriousness of the disease and more and more of them adhere to the treatment. Moreover, it is also interesting to evidence that it is not the financial restrictions that lead to cessation of the anti-osteoporotic treatment, but other conditions, independent of the financial ones. As an overview, it can be noticed, inclusively at the level of the doctors that dealing with their patients is insufficient as long as only half of those under corticosteroid treatment (a treatment known by the doctors probably as the greatest cause of osteoporosis) have a correct anti-osteoporotic treatment. 

**Conflict of interest**

None. 

The article is part of the “Introduction” and “Special Part” of the doctoral thesis of the corresponding author, Abobului Mihai, MD, PhD student. 
